# Noncoding RNAs and Human Induced Pluripotent Stem Cell-Derived Cardiomyocytes in Cardiac Arrhythmic Brugada Syndrome

**DOI:** 10.3390/cells12192398

**Published:** 2023-10-03

**Authors:** Benjamin Theisen, Austin Holtz, Viswanathan Rajagopalan

**Affiliations:** 1 Department of Biomedical and Anatomical Sciences, New York Institute of Technology College of Osteopathic Medicine at Arkansas State University, Jonesboro, AR 72401, USA; 2 Arkansas Biosciences Institute, Jonesboro, AR 72401, USA

**Keywords:** Brugada, noncoding, stem cells, heart, arrhythmia, ion channel, MiRNA, LncRNA

## Abstract

Hundreds of thousands of people die each year as a result of sudden cardiac death, and many are due to heart rhythm disorders. One of the major causes of these arrhythmic events is Brugada syndrome, a cardiac channelopathy that results in abnormal cardiac conduction, severe life-threatening arrhythmias, and, on many occasions, death. This disorder has been associated with mutations and dysfunction of about two dozen genes; however, the majority of the patients do not have a definite cause for the diagnosis of Brugada Syndrome. The protein-coding genes represent only a very small fraction of the mammalian genome, and the majority of the noncoding regions of the genome are actively transcribed. Studies have shown that most of the loci associated with electrophysiological traits are located in noncoding regulatory regions and are expected to affect gene expression dosage and cardiac ion channel function. Noncoding RNAs serve an expanding number of regulatory and other functional roles within the cells, including but not limited to transcriptional, post-transcriptional, and epigenetic regulation. The major noncoding RNAs found in Brugada Syndrome include microRNAs; however, others such as long noncoding RNAs are also identified. They contribute to pathogenesis by interacting with ion channels and/or are detectable as clinical biomarkers. Stem cells have received significant attention in the recent past, and can be differentiated into many different cell types including those in the heart. In addition to contractile and relaxational properties, BrS-relevant electrophysiological phenotypes are also demonstrated in cardiomyocytes differentiated from stem cells induced from adult human cells. In this review, we discuss the current understanding of noncoding regions of the genome and their RNA biology in Brugada Syndrome. We also delve into the role of stem cells, especially human induced pluripotent stem cell-derived cardiac differentiated cells, in the investigation of Brugada syndrome in preclinical and clinical studies.

## 1. Introduction

### 1.1. Sudden Cardiac Death

Sudden cardiac arrest (SCA) leading to sudden cardiac death (SCD) is a much more common cardiovascular (CV) cause of death in the young than it is in the elderly population [1,2,3]. SCD is relatively rare when compared to other CV causes of death, with the incidence between 1.3 and 3.2 per 100,000 person-years [1]. Most SCDs in young individuals aged 1–35 years are caused by potentially inherited heart diseases, including primary arrhythmogenic disorders, arrhythmogenic cardiomyopathy, hypertrophic cardiomyopathy, and dilated cardiomyopathy. Although such conditions remain a common cause of SCD at least until the age of 50 years, the most common cause of SCD beginning at 35 years of age is attributed to coronary artery disease [4]. While specific triggers for SCD have been identified (e.g., fever, sleep, exercise, acute arousal, rest, etc.), these events are often unexpected and unexplained even after autopsy. The majority of unexplained causes are attributed to sudden fatal arrhythmias [5]. Therefore, their true prevalence continues to be elucidated, though complete data are generally lacking. These arrhythmias are often genetically linked and can be broadly classified either as (i) defects in ion channels (channelopathies), or (ii) structural defects of the heart, both of which ultimately lead to improper conduction of electrical impulses through the myocardium [2].

Cardiac ion channelopathies are generally considered inherited primary electrical disorders in the setting of no apparent structural heart defects. These include Brugada syndrome (BrS), long QT syndrome, short QT syndrome, and catecholaminergic polymorphic ventricular tachycardia (VT) [6,7]. These four syndromes have been hypothesized to have in common an amplification of the spatial dispersion of repolarization, resulting in the development of polymorphic VT and ventricular fibrillation (VF) [8]. These channelopathies have been shown to present with an estimated prevalence of 1:2000 [2,9,10]. Although there are well-described genetic mutations, these conditions suffer from incomplete penetrance (proportion of individuals who possess a particular genotype and exhibit the expected clinical phenotype) and variable expressivity (series of signs and symptoms that can occur in different people with the same genetic condition) [5,11,12]. The rarity, combined with challenging diagnosis and unforeseen fatal natural history, makes these conditions both important and urgent to study [1]. Among these disorders, the BrS represents a complex clinical problem, and the pathophysiological basis is incompletely understood and likely heterogeneous in nature [6].

The aim and objective of this invited review is to discuss the current knowledge on the role of noncoding RNAs (ncRNAs) and stem cells (both expanded later) in the cardiac arrhythmic BrS. Although both have revolutionized the understanding of cellular and molecular biology, their roles and interactions in BrS are unclear and deserve focused attention and further elucidation.

The review will open with a background on clinical and pathological aspects of the BrS. Subsequently, it will focus on our current understanding of BrS-related mechanisms involving noncoding and stem cell biology. We will also discuss the potential roles of ncRNAs in disease pathogenesis and as biomarkers in BrS. Furthermore, the study of ncRNAs in a stem cell-based vitro model of BrS will be discussed.

### 1.2. Brugada Syndrome (BrS)

#### 1.2.1. Epidemiology and Presentation

The prevalence of adult BrS with a spontaneous type 1 (discussed below) electrocardiogram (ECG) is estimated at 0–0.1% in the US and Europe and as high as 0.94% in Asian countries. Southeast Asian countries show the highest prevalence of about 3.7 per 1000 [13,14,15,16,17,18]. About 80–90% of diagnosed cases exhibit a male predominance [1,9,19]. BrS is attributed to 4–12% of all SCDs, and up to 28% of SCDs in patients with apparently macrostructurally normal hearts [15,20]. The median age is 45 (35 to 55) years, and there is a very low prevalence in children (about 1:20,000). The vast majority (94%) of the patients are 16 to 70 years old [21]. Approximately two-thirds of the patients are asymptomatic and one-third can present with syncope [22,23,24,25]. The clinical manifestations of BrS can be precipitated by multiple factors such as fever, alcohol, and a variety of drugs [26,27,28]. In addition, SCD and arrhythmias typically occur during rest, sleep, or following large meals, associated with episodes of vagal predominance and/or bradycardia [29,30,31].

A BrS electrocardiogram (ECG) can present in three possible types. The typical type I ECG presents with coved-type ST-segment elevation of ≥2 mm in right precordial leads V_1_ and V_2_ (second, third, or fourth intercostal spaces). This is followed by terminal negative T wave and high J point (distinctive deflection at the QRS-ST junction). In other words, type I can be characterized by J-point elevation ≥ 2 mm with descending ST segment and negative T wave in the right precordial leads V1 and V2 [32]. Type 2 has a saddle-back-shaped ST segment in lead V2 with a J-point elevation of ≥2 mm and a terminal ST-segment elevation (≥1 mm), followed by a positive T wave. Type 3 has a saddle-back type in lead V2 with a J-point elevation of ≥2 mm and a terminal ST-segment elevation of <0.1 mm [33]. The type 2/3 patterns may present with variable levels of ST-segment elevation. In patients presenting with Type 2 or Type 3 ECG at baseline, a Type 1 BrS-ECG may be unmasked by provocation testing with sodium channel blockers such as procainamide, ajmaline, flecainide, or pilsicainide. Although the recently developed Shanghai score recommends additional information (clinical history, family history, and/or genetic testing results) to make a clearer diagnosis, there is not a clear consensus regarding whether a drug- or fever-induced type 1 pattern is diagnostic [9,34]. In addition to the ventricles, BrS can also be associated with spontaneous atrial arrhythmias from 6% to 38% and inducible atrial arrhythmias from 3% to 100% [35]. Morita et al. reported spontaneous atrial fibrillation in about 39% of the BrS patient population [36]. Furthermore, BrS is also vulnerable to being misdiagnosed for right bundle branch block, potentially subjecting the patient community to a higher risk of mortality and/or morbidity [37,38]. Multiple conditions have been associated with BrS. These include hypothyroidism, thyroid storm secondary to Graves’ disease, drugs, sympathovagal imbalance, metabolic and electrolyte abnormalities, cancer, diabetes mellitus, testosterone, schizophrenia, epilepsy, etc. [28,39,40,41,42,43,44,45,46,47].

The major goal of treatment for BrS patients is sustained prevention of SCA/SCD events. This is primarily accomplished via on-demand therapeutic implantable cardioverter–defibrillator devices, i.e., ICDs, whether transvenous or subcutaneous [48,49]. This can be life-saving, particularly in those who had previous documented episodes of SCA or VT, who have demonstrated inducible ventricular fibrillation (VF), or who have a history of syncope that is deemed likely due to arrhythmic events (e.g., ECG changes). In patients with recurrent ICD shocks that cannot be managed with medical therapy (e.g., quinidine), or in those in whom an ICD was not implanted, despite being indicated (e.g., strong patient preference), radiofrequency ablation helps in the treatment. Dr. Brugada, Dr. Pappone and team [50,51] showed that radiofrequency substrate ablation in BrS patients identified in the presence of flecainide could eliminate the BrS phenotype. They also showed that radiofrequency ablation is effective in potentially eliminating arrhythmic consequences via BrS ECG normalization and VT/VF noninducibility. More recently, Nademanee et al. [52] performed a long-term follow-up study for 48 months in 159 BrS patients using the BRAVO registry. The results revealed that 81% and 96% showed freedom from VF recurrence after a single ablation and following repeat ablations, respectively. Future studies are expected to investigate the potential for ablation to serve as an alternative to ICD in symptomatic BrS, and also its efficacy in asymptomatic BrS.

On the other hand, Brugada Phenocopy [53,54,55] (BrP) is a clinical condition similar to the authentic congenital BrS. Although controversial [56], patients with BrP usually do not suffer BrS-related symptoms or have a negative drug challenge, and they exhibit an unknown risk of ventricular arrhythmias/SCD. BrP patients present with type 1 or type 2 electrocardiographic patterns similar to BrS. They are elicited only under transitory pathophysiological conditions (including myocardial ischemia, myopericarditis, ionic abnormalities, hypothermia, etc.), with normalization/disappearance of the ECG patterns in the days after resolution of those conditions or disappearance of the trigger factors. It is possible that omics [57] approaches may better differentiate BrS from BrP. For the purposes of this review, we will essentially focus on BrS.

#### 1.2.2. BrS Genetics

BrS was initially considered to be inherited in a monogenic, autosomal dominant fashion. However, it is now also considered to potentially encompass an oligogenic or polygenic inheritance, in which multiple ‘genetic modifiers’ either exacerbate or alleviate the phenotypic expression of the primary genetic defect [58]. Nonetheless, the genetics of BrS remain unclear in the majority of clinically confirmed cases. Several hundreds of pathogenic variants in multiple genes have been linked to BrS. It is important to note that *SCN5A* is recognized as a causative gene for BrS [59]. It is also imperative to understand that *SCN5A* is associated with a more severe disease phenotype of BrS [60]. On the other hand, although the role of other genes may be associated with the disease, they are not yet considered causative.

Based on pathophysiological mechanisms [16], the BrS-related genes can be classified based on whether they affect:

(i) Sodium current (I_Na_): *FGF12*, *GPD1L*, *PKP2*, *RANGRF*, *SCN1B*, *SCN2B*, *SCN3B*, *SCN5A*, *SCN10A*, *SLMAP*, *TRPM4*.
(ii) Potassium current (I_K_): *ABCC9*, *KCND2*, *KCND3*, *KCNE1L*, *KCNE3*, *KCNE5*, *KCNH2*, *KCNJ8*, *SEMA3A*.
(iii) Calcium current (I_Ca_): *CACNA1C*, *CACNA2D1*, *CACNB2B*.
(iv) Miscellaneous/Other: *HCN4*, *HEY2*, *LRRC10*.

Variants in these genes may explain about 30–35% of the cases; however, the rest of the cases remain genetically unsolved (phenotype-positive and genotype-negative). Although most of the BrS mutations are associated with a loss-of-function effect, variants in *KCND3*, *KCNE3* and *KCNE5* genes result in a gain-of-function effect [11,61]. BrS may not be a pure Mendelian disorder, but rather ECG patterns resulting from multifactorial molecular abnormalities. Dissecting these mechanisms is critical to make progress in BrS diagnosis and management [17,62].

#### 1.2.3. BrS Arrhythmogenic Mechanisms

BrS could be considered a predominant right ventricular (RV) disease associated with J waves (J-wave syndrome with similarities to ERS). This presents a more prominent I_to_-mediated action potential notch in the RV epicardium and reduced expression of sodium channels and gap junctions. The two proposed major mechanistic hypotheses are as follows [1,15,63,64].

The repolarization hypothesis proposes that an outward shift in the balance of currents at the end of phase 1 of the RV epicardial action potential resulting from a genetically mediated imbalance in ionic currents generates repolarization anomalies. The mutations in the genome typically result in either decreasing the inward depolarizing Na^+^ and Ca^++^ currents or increasing the outward repolarizing K^+^ currents. These result in an outward shift in the balance of current active during the early phases of the action potential, thereby increasing the risk of arrhythmia and SCD [65]. These abnormalities could also lead to the development of reentry during phase 2 of the action potential. These, in turn, could generate closely coupled premature beats, which can trigger polymorphic VT or VF. On the other hand, the depolarization hypothesis proposes that fibrosis and reduced expression of sodium channels and gap junction proteins (e.g., connexin 43) in the RV outflow tract could lead to dispersed conduction, fractionated electrograms and eventual BrS ECG phenotype [66]. Neither of these hypotheses has been conclusively established as the sole underlying mechanism. On the other hand, both the hypotheses are not mutually exclusive and may be synergistic. Furthermore, a current-load mismatch mechanism has also been proposed [67,68,69,70].

Studies have viewed BrS as a condition presenting with functionally impaired conduction [71]. However, many studies have also considered BrS as a subepicardial disease [72,73,74,75,76,77], particularly as a subepicardial cardiomyopathy with subtle microstructural abnormalities of the extracellular matrix, especially within the RV subepicardial myocardium [72,78]. In addition to electrophysiological abnormalities, this is also mediated by abnormal distribution of actin and decreased focal adhesions [79]. Taken together, the electrocardiographic changes and the arrhythmogenic substrate are postulated to be mediated by genetic and environmental susceptibilities along with impaired ionic currents, precipitating a reduction in epicardial conduction reserve and facilitating current-to-load mismatch at sites of structural discontinuity [72].

### 1.3. Noncoding RNAs and Their Major Types

Only about 2% of the entire human genome (<5% of the mammalian genome) is protein-coding. Nonetheless, a large majority of the remainder of the genome (noncoding) is actively transcribed/transcribable [80,81,82]. Genome-wide association studies (GWAS) have also shown that most of the loci associated with electrophysiological traits are located in noncoding regulatory regions and are expected to affect gene expression dosage and cardiac ion channel function [74,83]. Based on the Encyclopedia of DNA Elements (ENCODE) project [84,85,86], we know that most disease-associated common variants are noncoding. The studies have also assigned biochemical functions for 80% of the genome, in particular outside of the well-studied protein-coding regions. In addition to areas that encode ncRNAs (discussed below), noncoding variants are located in cis-regulatory elements, including enhancers, promoters, and insulators [87]. Furthermore, more than two decades of research have shown that the ncRNAs serve an expanding number of regulatory and other functional roles within the cell, including and not limited to transcriptional, post-transcriptional, and epigenetic regulation [88].

Noncoding RNAs can be broadly classified as (1) primarily housekeeping ncRNAs and (2) regulatory ncRNAs. The primarily housekeeping ncRNAs include (1a) rRNA (ribosomal RNA), (1b) tRNA (transfer RNA), (1c) snRNA (small nuclear RNA), and (1d) snoRNA (small nucleolar RNA). The regulatory RNAs include (2a) short ncRNAs and (2b) and long ncRNAs (>200 nucleotides). The short ncRNAs include miRNA (microRNA), siRNA (small interfering RNA), piRNA (piwi-interacting RNA), and scaRNA (small cajal body-specific RNAs). The long ncRNAs include lncRNAs, eRNA (enhancer RNA), circRNA (circular RNA), and NAT (natural antisense transcripts). Among these, miRNAs and lncRNAs are known to be involved in BrS biology.

#### 1.3.1. MiRNAs

MicroRNAs (miRNAs) are small ncRNAs averaging ~22 nucleotides in length and have been identified as important mediators in regulating the expression of numerous coding genes [89,90]. Most miRNAs are processed into precursor miRNAs (pre-miRNAs) and mature miRNAs following transcription from DNA sequences into primary miRNAs. In general, the miRNAs interact predominantly with 3′ untranslated regions (UTRs) of target mRNAs to inhibit translation [91]. This is accomplished by either the 5p or 3p strands of the mature miRNA duplex loading into the Argonaute family of proteins to generate a miRNA-induced silencing complex, miRISC. The majority of the interactions between miRNA and miRNA response elements in animal cells are not fully complementary. Although a functional miRNA:miRNA response element interaction occurs via the 5′ seed region (nucleotides 2–8), additional pairing at the 3′ end supports the specificity and stability of the miRNA–target interaction. As miRISC binds to target mRNAs, translational inhibition is induced most likely by interfering with the eIF4F complex. Subsequently, m7G-decapped mRNAs may undergo 5′–3′ degradation via the exoribonuclease *XRN1* [90,92,93]. While most miRNAs are associated with downregulation, some studies have demonstrated miRNA-induced activation as well [94,95,96]. Friedman et al. [97] estimated that about two-thirds of the human protein-coding genes have target sites for miRNA in their 3′UTR. Among other targets in the cardiac tissue, miRNAs have been shown to play key roles in regulating ion channels necessary for normal physiologic cardiac function and rhythm [98].

miRNAs have been shown to regulate the differentiation and maturation of human embryonic stem cells to cardiomyocytes. *miR-1* (microRNA-1) was shown to facilitate the electrophysiological maturation of human stem cells [99]. Furthermore, *miR-200c* was found to be a repressor of differentiation and maturation of human stem cell-derived cardiomyocytes [100].

#### 1.3.2. Long Noncoding RNAs

In contrast to the short noncoding miRNAs, long noncoding RNAs (lncRNAs), as their name implies, are significantly lengthier molecules with >200 nucleotides [82]. LncRNAs are the largest population of ncRNAs, and human lncRNA genes exceed protein-coding genes by about 4.8-fold [101]. They are classified based on their position, subcellular localization, and function. LncRNAs can be described as those that span multiple introns or exons (sense lncRNAs), are located within an intron (sense intronic lncRNAs), are transcribed from genomic regions that are intergenic (long intergenic lncRNAs or lincRNAs), are located in the enhancer region, and are antisense to another gene (AS-lncRNAs). LncRNAs can also be characterized by their interactions with proteins, other RNAs, and DNA and their capacities to be guides, to tether protein complexes (scaffolds), and to block binding to their targets (decoys) and competing endogenous RNAs (ceRNAs), e.g., to bind and inactivate miRNAs [88] (sponge). The nuclear-localized lncRNAs may recruit chromatin-modifying enzymes, interact with transcription factors, or regulate splicing. The cytoplasmic lncRNAs may regulate gene expression post-transcriptionally by acting as miRNA decoys or via modulation of translation and mRNA stability. Individual lncRNAs are not necessarily limited to any one specific function; many play multiple roles within the cell. In addition, lncRNAs have been shown to act in either a *cis* (enhancer and promoter activities) or *trans* (on distal chromosomal sites; transcription factor binding) fashion [102,103,104,105]. Although lncRNA sequences are not as conserved as miRNAs across species, studies indicate conservation of lncRNA structure and function [106,107]. LncRNAs may be either linear (more widely studied) or circular (circRNAs), and both play important roles in CV disorders [82,88,108,109].

#### 1.3.3. Stem Cells, Human Induced Pluripotent Stem Cells Differentiated into Cardiomyocytes and BrS

Stem cells are unspecialized cells that exhibit characteristics enabling them to multiply themselves and/or transform into various forms of mature cells, including those of the heart. They can be classified into totipotent, pluripotent, multipotent, oligopotent and unipotent stem cells [110]. Among these, the pluripotent stem cells can form cells of all the germ layers and non-extraembryonic structures.

BrS has been reported after autologous peripheral blood stem cell transplantation in a 74-year-old male with acute myeloid leukemia [111]. The role of stem cells in the pathogenesis of BrS is not fully clear. Nonetheless, studies in the recent past have largely focused on using induced pluripotent stem cells (iPSCs) for developing models for BrS research [112]. Adult somatic cells from mice [113] and humans [114] have been inducibly reprogrammed into pluripotent cells using four transcription factors, *Oct3/4*, *Sox2*, *Klf4*, and *c-Myc*. Human iPSCs differentiated into cardiomyocyte-like cells (hiPSC-CMs) possess unique characteristics of beating heart cells along with the advantage of rapid culture in the laboratory with adult cells obtained from individual patients (personalized medicine). Multiple congenital heart diseases have been investigated using hiPSCs-CMs [115,116,117]. A recent study showed that Wnt/β-catenin signaling inhibited Na_v_1.5 expression in both male and female hiPSC-CMs. In addition, inhibition of Wnt/β-catenin signaling upregulated Na_v_1.5 in BrS iPSC-CMs via both transcriptional and posttranscriptional mechanisms [118]. HiPSC-CMs have become an increasingly important tool for studying BrS [119].

## 2. Noncoding RNAs and Stem Cells in BrS

NcRNAs have been shown to regulate cellular reprogramming, pluripotency, cardiac proliferation, differentiation and maturation. Modulation of ncRNAs may enhance the quality and quantity of stem cells and their derivatives for potential clinical application in cardiac patients [120,121]. However, the role of ncRNA-mediated regulation of stem cells in BrS is unclear and deserves future attention.

Barajas-Martinez et al. [122] showed that the BrS/early repolarization syndrome (ERS) phenotype was found in hiPSC-CMs, and more specifically, only in the proband carrying all three mutations of *SCN9A*, *PXDNL*, and *FKBP1B* variants, and not one or two alone [58]. These findings support a polygenic cause of the BrS arrhythmic phenotype. Al Sayed et al. [123] presented a specific cellular electrophysiological phenotype common to all BrS hiPSC-CM lines with various genetic backgrounds. This included high early after-depolarizations associated with an abnormal increase in *I*_Na,L_, and correlated with corresponding patients’ ECG *J*-point elevation. However, they focused on the coding regions of select genes known to be associated with BrS. Studies need to expand to the entire noncoding compartment.

**Figure 1:**
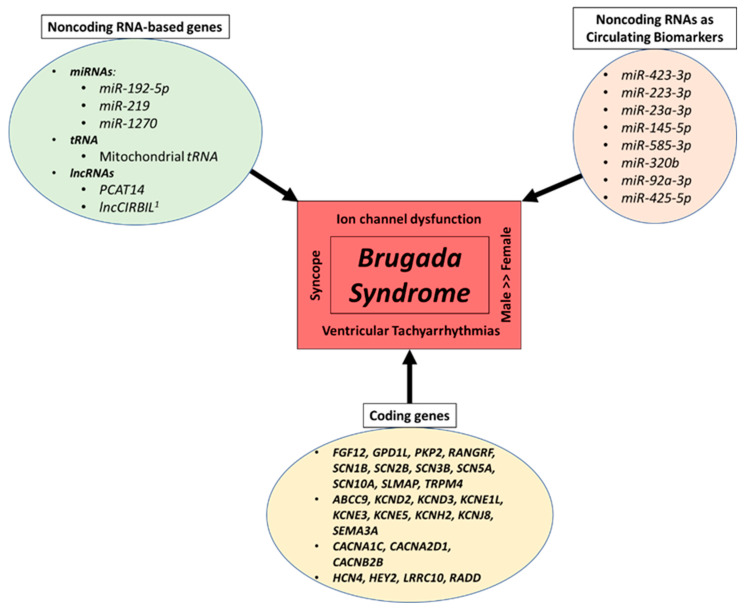
Major coding genes, noncoding RNA-based genes and noncoding RNAs as circulating biomarkers related to BrS. The figure outlines the major BrS-related coding genes, BrS-related noncoding RNA-based genes, and BrS-related noncoding RNAs as circulating biomarkers. It also highlights select major pathophysiological hallmarks of BrS. miRNA—microRNA; lncRNA—long noncoding RNA; tRNA—transfer RNA; ^1^ preprint.

In the following sections, we will discuss the BrS genes listed in Section 1.2.2 above that have been implicated with ncRNAs and stem cells. This will be approached from the perspective of noncoding biology studied using various models including hiPSC-CMs. Major BrS-related coding genes, BrS-related ncRNA-based genes and BrS-related ncRNAs as circulating biomarkers are presented in Figure 1.

### 2.1. SCN5A

*SCN5A* encodes the alpha subunit of the principal voltage-gated cardiac Na channel protein, which predominates the cardiac sodium current *I*_Na_. This current underlies the fast upstroke of the cardiac action potential [124]. The *SCN5A* is causative and is one of the most commonly implicated genes in BrS, ranging from about 15% to 30% of known cases [14,15,16,17,18,125,126,127]. Single mutations in *SCN5A* have been shown to be inadequate in leading to clinical BrS [128]. Therefore, the genetic background may contribute to the pathophysiology of BrS [129]. In addition, compound *SCN5A* mutations may cause more severe clinical and electrocardiographic manifestations including BrS [130]. Female patients with BrS are much rarer, display less type 1 Brugada ECG, and exhibit lower inducibility rates than males. However, female patients with BrS with arrhythmic events exhibit higher *SCN5A* mutation rates, and a relationship between gender vs. age at the onset of arrhythmic events and ethnicity [131]. In the majority of patients referred for BrS genetic testing with a single *SCN5A* mutation (74%), this was localized to the transmembrane region of the channel [127,132].

Park et al. [133] studied genetic variants in the *SCN5A* promoter in a large kindred that exhibited a mixed phenotype (BrS and AV conduction disease) and marked variation in phenotype severity. The *SCN5A* promoter is known to span an ~2.8 kb segment of DNA that extends into intron 1 of *SCN5A* and includes 2.1 kb of the 5′ upstream sequence of exon 1, exon 1 (142 bp and noncoding), and the proximal intron 1 regions that are relatively GC-rich (60.6% GC content) [134]. They found that the promoter variants (*rs41310749* and *rs41310239*) were significantly associated with disease severity—mild vs. severe phenotype. Bezzina et al. [135] studied 2.8 kb of *SCN5A* promoter (including noncoding regions) and identified a haplotype variant consisting of six polymorphisms in near-complete linkage disequilibrium. This occurred at an allele frequency of 22% in Asian subjects and was absent in white and black subjects. They concluded that genetically determined variable human cardiac sodium channel transcription is associated with variable conduction velocity, an important contributor to arrhythmia susceptibility. Yagihara et al. [136] resequenced the *SCN5A* core promoter region and the regulatory regions of *SCN5A* transcription in 1298 patients with arrhythmia phenotypes, which included 583 BrS patients. They identified 26 novel rare variants in the *SCN5A* promoter in 29 patients affected by various arrhythmias, which included 14 BrS patients. By employing luciferase reporter assay, they functionally characterized 3 BrS variants, which displayed decreased promoter activity compared to wild type. Studies have also indicated that heteroplasmic mutations in mitochondrial tRNA genes might alter protein translation associated with the respiratory chain, possibly contributing to BrS [137].

Multiple microRNAs were shown to regulate *SCN5A* either directly (*miR-98*, *miR-106*, *miR-200*, and *miR-219*) or indirectly (*miR-125* and *miR-153*). Some of the microRNAs were also functionally validated as regulators of *SCN5A* in vitro and/or in vivo. Most *SCN5A* variants in a BrS family were localized in noncoding regions, implicating an impact on the miRNA–target complementarities. These mutations were shown to form a new *miR-1270* binding site, and *miR-1270* overexpression displayed a significant reduction in *SCN5A* expression. The study also identified *hsa-miR-219a-rs107822* (*hsa-miR*: Homo sapiens microRNA) [138]. Furthermore, the group showed that *miR-219* increased *SCN5A*/Nav1.5 transcript and protein expression and increased sodium current. In vivo administration of *miR-219* abolished select effects of flecainide intoxication in mice (QRS prolongation) [139]. Knockdown of *miR-200c* led to a significant increase in the expression of *SCN5A* in cardiomyocyte lineage cells differentiated from human embryonic pluripotent stem cells [100]. *SCN5A* polymorphism H558R was reported to be a potential modifier that protects against VF occurrence in BrS. This was mediated by decreased cardiac *SCN5A* promoter methylation and an increase in the *SCN5A* expression levels [140]. *SCN5A*-R1193Q was also shown to be associated with cardiac conduction disturbances and defibrillator shocks (appropriate) in BrS patients [141]. The minor allele frequency of T to C variant polymorphism in the 3′-UTR of the *SCN5A* gene, which was predicted to be adjacent to the potential binding site of *miR-192-5p*, was found in the BrS population at a rate higher than the controls. *miR-192-5p* significantly downregulated expression of *SCN5A*, Nav1.5 and peak sodium current density I_Na_ generated by Nav1.5. Accordingly, in left atrial appendage samples from patients with atrial fibrillation, *miR-192-5p* expression levels were upregulated, which was associated with downregulation of *SCN5A*/Nav1.5 [142].

Studies using patient-specific hiPSC-CMs have successfully recapitulated BrS phenotypes such as blunted inward sodium current, increased triggered activity, and abnormal Ca^2+^ handling. This includes increased arrhythmogenicity in both asymptomatic and symptomatic R1913C *SCN5A* mutation carriers, and also pro-arrhythmic changes in Na channel function not detected using conventional heterologous expression systems [143,144,145,146]. The Wu group showed that BrS hiPSC-CMs displayed reductions in inward sodium current density and reduced maximal upstroke velocity of action potential compared with control hiPSC-CMs. In addition, BrS hiPSC-CMs demonstrated an increased burden of triggered activity, abnormal calcium (Ca^2+^) transients, and beating interval variation. Correction of the causative *SCN5A* variant (*rs397514446*) via genome editing in hiPSC-CMs showed resolution of triggered activity and abnormal Ca^2+^ transients. BrS hiPSC-CM gene expression also correlated with gene expression from BrS human cardiac tissue [143]. BrS patient-specific hiPSC-CM with *SCN5A* c.1100G>A, leading to Na_v_1.5_p.R367H mutation, recapitulated loss of function of sodium channel current including pro-arrhythmic changes in channel function not detected using conventional heterologous expression systems [144]. HiPSC-CM from BrS patients carrying a heterozygous *SCN5A* mutation p.S1812X also recapitulated multiple clinical phenotypical characteristics. These included reduction in *I*_Na_ density and Na_V_1.5 expression, impaired localization of Na_V_1.5 and connexin 43 (Cx43) at the cell surface, reduced action potential upstroke velocity and conduction slowing, and also Phosphodiesterase 3 blocker (cilostazol and milrinone)-induced inhibition of *I*_to_ and alleviation of arrhythmic activity [147]. HiPSC-CMs derived from a BrS patient with a compound *SCN5A* mutation (p. A226V and p. R1629X) and another BrS patient with a milder p. T1620M mutation revealed that severe *I*_Na_ deficiency could lead to remodeled baseline action potentials vulnerable to heart rate-induced, *I*_to_-sensitive proarrhythmic increased phase-1 repolarization changes [145]. In a recent study [148], the authors investigated a rare BrS familial variant NM_198056.2:c.3673G>A (NP_932173.1:p.Glu1225Lys) using hiPSC-CMs. A lentiviral vector encoding a GFP-tagged *SCN5A* gene carrying the BrS variant showed impairment of the mutated Nav1.5. This included a reduction in peak sodium currents in hiPSC-CMs overexpressing the p.E1225K Nav1.5 channel. Another recent study investigated the *SCN5A* variant (c.3148G>A/p.Ala1050Thr) causing loss of function of sodium channels. They reported increased channel sensitivity to high temperature and lipopolysaccharide challenge in hiPSC-CMs from a BrS cell line with this variant but not in two non-BrS hiPSC-CM lines. They also suggested that lipopolysaccharides may exacerbate the BrS phenotype by enhancing autophagy, whereas fever may exacerbate the BrS phenotype by inhibiting PKA signaling in BrS cardiomyocytes [149]. *SCN5A*-1795insD leads to an overlap syndrome, in which patients present with both features of BrS and cardiac conduction disease (decreased *I*_Na_) and long QT syndrome type 3 (increased late *I*_Na_). Recently, Nasilli et al. showed that chronic mexiletine incubation in *SCN5A*-1795insD hiPSC-CMs followed by wash-out increased peak *I*_Na_, action potential upstroke velocity and duration [150]. Additional models of BrS are also available using hiPSC-CMs. These include two cell lines with mutations at *SCN5A* c.53506 G>A and c.2102 C>T, and others at *SCN5A* c.392 + 3A > G splice-site variant and *SCN5A* p.D356Y (classified by the American College of Medical Genetics and Genomics as “likely pathogenic” or “variant of undetermined significance”) [151,152,153].

In addition to BrS, *SCN5A* mutations have been associated with other inherited arrhythmia syndromes such as Long QT3 and ERS, and a variety of conduction disorders such as sick sinus syndrome [15]. Whether ncRNAs play roles in distinguishing the phenotypes needs to be closely investigated.

### 2.2. SCN1B and SCN10A

In addition to *SCN5A*, other genes that code for sodium channel subunits have also been observed to be linked with BrS. Specifically, *SCN1B*, *SCN2B* and *SCN3B* code for sodium channel beta subunits and can lead to electrical dysfunction seen in BrS. In the study by El-Battrawy et al., two variants (c.629T>C/p.L210P and c.637C>A/p.P213T) were detected in *SCN1B* using a hiPSC-CM model. The peak I_Na_, late I_Na_ and amplitude and upstroke velocity of action potentials were decreased in hiPSC-CMs from BrS patients with the mutant *SCN1B* [112,154]. While ajmaline reduced amplitude and Vmax of action potential and exhibited a stronger blocking effect in hiPSC-CMs of BrS compared to controls, carbachol increased arrhythmia events and the beating frequency in BrS [112,154,155]. In another study [156] by the same group, hiPSC-CMs were created from a BrS patient (diagnosed via ajmaline) with two variants within *SCN10A* (p.Arg1250Gln and p.Arg1268Gln2). They showed a significant reduction in I_Na_ as well as amplitude and upstroke velocity of action potentials. In addition, ajmaline’s effects on action potentials were stronger in BrS-hiPSC-CMs than in the controls.

van den Boogaard et al. [157] demonstrated that the *rs6801957* SNP is located in the *SCN10A* intronic enhancer, and this SNP is associated with QRS prolongation and in linkage disequilibrium with variants associated with BrS. This SNP also modulates T-box factor binding and activity of the isolated enhancer fragment. They also subsequently reported a direct correlation of *SCN5A* expression with the presence of the *rs6801957* risk-associated SNP in the *SCN10A* intronic enhancer in humans [158]. Bezzina et al. [159] also conducted a GWAS of 312 individuals with BrS and 1115 controls. They reported that their strongest association (*rs10428132*) resided in intron 14 of the *SCN10A* gene, which is located adjacent to *SCN5A* on chromosome 3p21-22. This SNP contained a nonsynonymous variant affecting *SCN10A* (*rs6795970*, *r*^2^ = 0.97 with *rs10428132*). The haplotype tagged by this SNP has been associated with variability in PR interval and QRS duration in the general population [160,161,162,163]. Furthermore, the SNP associated with the signal at 6q22 tagged a linkage disequilibrium block that entirely encompasses the *HEY2* gene and extends into the first intron of *NCOA7* (*ERAP140*). Behr et al. [164,165] conducted a multi-center study and sequenced seven candidate genes (*SCN10A, HAND1, PLN, CASQ2, TKT, TBX3,* and *TBX5*) in 156 Caucasian *SCN5A*-mutation-negative BrS patients. Eighty percent were male, with a mean age of 48, and had symptoms (64%) and/or a family history of sudden death (47%) or BrS (18%). They found that the common intronic SNP *SCN10A* V1073 was strongly associated with BrS and demonstrated loss of NaV1.8 function.

More recently, Man et al. [166] investigated the cardiac expression of *SCN10A* and the function of a variant-sensitive intronic enhancer known to be linked to the regulation of *SCN5A*. Using a CRISPR/Cas9 genome editing mouse model, they reported that the enhancer function and expression of a cardiac-specific *SCN10A-short* transcript are modulated by genetic variants in and around *SCN10A*. They also proposed that the transcriptional regulation of a functional C-terminal portion of cardiomyocyte Na_V_1.8 is modulated by noncoding genetic variation. This impacts Na_V_1.5 function, cardiac conduction velocities, and arrhythmia susceptibility. A recent study has shown that the *SCN10A* gene (Na_V_1.8) contributes to I_NaL_ formation in human atrial cardiomyocytes, and CRISPR/Cas9 deletion of this gene modulates proarrhythmogenic triggers in human atrial cardiomyocytes [167].

### 2.3. SCN3B

*SCN3B* is a sodium voltage-gated channel beta subunit 3 and has been found to be important for the intracellular trafficking of *SCN5A* and preservation of sodium currents based on a study [168] of 181 BrS patients. Another study [119,169] showed that *SCN3B* is expressed in hiPSC-CMs, and knockdown of *SCN3B* in mixed-phenotype LQTS3/BrS hiPSC-CMs (with E1784K *SCN5A* mutation) successfully unmasked the phenotype of BrS. Furthermore, isogenic control of LQTS3/BrS (corrected-LQTS3/BrS) hiPSC-CMs gained normal electrophysiological properties.

### 2.4. CACNB2

The link in the cardiac excitationcontraction coupling is calcium (Ca^2+^), which starts during the upstroke of the action potential. This causes the opening of the L-type voltage-dependent Ca^2+^ channels, which are composed of protein complexes of multiple subunits each. The pore-forming Ca_v_1.2 α_1_-subunit is encoded by the *CACNA1C* gene and the β-(Ca_v_β2b) subunit by the *CACNB2* gene [98,170]. A variant in either of the subunits may predispose an individual to BrS. Both the genes have a prevalence of 1–2% in BrS cases [16,171].

In a recent study [172], hiPSC-CMs were generated from a BrS patient who presented with recurrent SCAs. He had a missense variant of uncertain variance found in the *CACNB2* gene (c.425C>T/p.S142F). The authors reported that the BrS-hiPSC-CMs had reduced peak L-type calcium current and *CACNB2* protein expression. They also had increased arrhythmia-like events at baseline, which were suppressed by quinidine and bisoprolol. In another study using hiPSC-CMs, calcium voltage-gated channel auxiliary subunit beta 2 (*CACNB2*) variant c.1439C>T/p.S480L was found to be associated with short QT syndrome 5 (SQTS5) phenotype overlapping with BrS. Amiodarone (and not quinidine) demonstrated a significant antiarrhythmic effect [173].

### 2.5. KCND2

J-wave syndromes such as the BrS are characterized by distinctive electrocardiographic J waves (discussed earlier) and are associated with an increased risk of life-threatening ventricular arrhythmias. A recent preprint [174] reported that knockout of *lncCIRBIL* increased the frequency of J waves and the susceptibility to ventricular arrhythmias in mice with the transmural difference in *KCND2.* In addition, I_to_ currents selectively increased in the right ventricle, but not the left ventricle. In contrast, cardiomyocyte-specific transgenic overexpression of *lncCIRBIL* developed the opposite effects. In addition, human homologous conserved fragment of *lncCIRBIL* reduced I_to_, downregulated action potential notch and prolonged action potential duration measured at 20% repolarization in hiPSC-CMs.

### 2.6. GPD1L

*GPD1L* refers to the glycerol-3-phosphate dehydrogenase 1-like gene and catalyzes the conversion of sn-glycerol 3-phosphate to glycerone phosphate [175]. Makiyama et al. [176] screened 80 unrelated Japanese BrS patients for *GPD1L* mutations. They reported one synonymous mutation as well as one intronic variant, both of which were absent in 220 control alleles.

### 2.7. PKP2

Plakophilin proteins contain several armadillo repeats and localize to cellular desmosomes and nuclei. They also participate in linking cadherins to intermediate filaments in the cytoskeleton. Cerrone et al. [177] investigated *PKP2* variants in the genomic DNA of 200 BrS patients with no signs of arrhythmogenic cardiomyopathy, and no mutations in select BrS-related genes (*SCN5A, CACNa1c, GPD1L*, and *MOG1*). They found 2.5% of cases with missense plakophilin 2 (*PKP2*) mutations. Furthermore, they reported that hiPSC-CMs with a PKP2 deficit drastically reduced *I*_Na_. In addition, the deficit was restored by transfection of wild type but not BrS-related *PKP2*.

### 2.8. RADD

Belbachir et al. [79] used whole-exome sequencing in a three-generation pedigree with five BrS-affected members. This allowed the identification of one rare non-synonymous substitution (p.R211H) in *RRAD*. This gene encodes the RAD GTPase and was carried by all affected members of the family. The study revealed that BrS hiPSC-CMs expressing the *RRAD* variant recapitulated single-cell electrophysiological features of BrS, including altered Na+ current and cytoskeleton disturbances.

### 2.9. HEY

*HEY* genes act as transcriptional repressors downstream of the Notch signaling and encode a family of basic helix–loop–helix (belch) proteins. *HEY2* CC genotype (downstream noncoding site) may be a prognostic marker for BrS, protectively acting to prevent VF presumably by regulating the repolarization current [178,179]. In addition, among the six mutations within *SCN5A* in 784 patients with BrS, one was a splice-site mutation, i.e., 3840 + 1 G>A [Intron 21].

### 2.10. Others

A recent metagenomic study with whole-genome sequencing of blood samples from 100 control subjects and 100 type 1 BrS patients identified the lncRNA *PCAT14* (prostate cancer-associated transcript 14) region as a potential candidate [180]. More studies are needed to investigate the involvement of lncRNAs in BrS biology and clinical translation. Using whole genome and transcriptome sequencing of post-mortem FFPE tissues, Andersen et al. [181] investigated DNA variants in SCD victims with macrostructurally normal hearts. They detected 23 candidate variants in regulatory sequences of cardiac genes. These included a variant in the promoter region of *NEXN*, c.-194A>G, which was significantly associated with decreased expression of *NEXN* and cardiac hypertrophy. It is not clear if this is directly associated with BrS, and it needs to be studied in this patient population.

### 2.11. Noncoding RNAs as Potential BrS Clinical Biomarkers

While mass-spectrometry-based protein biomarkers [182,183] have been explored, Ikeuchi et al. [184] employed 3D-Gene Human miRNA microarray/Oligo Chip in 70 patients with BrS and compared them with 34 control individuals. The results revealed >3-fold upregulation of eight circulating miRNAs and downregulation in one circulating miRNA. *Hsa-miR-423-3p*, *hsa-miR-223-3p*, and *hsa-miR-23a-3p* were independently associated with BrS based on multivariate logistic regression analysis (*p* < 0.0001). In a recent study of 21 definite BrS patients, Steinberg et al. [185] showed that a distinct miRNA expression profile was observed from total RNA extracted from peripheral blood compared with unaffected control individuals. Together, 38 miRNAs were upregulated and 4 were downregulated. In addition, miRNAs such as *miR-145-5p* and *miR-585-3p* were associated with the symptomatic status of BrS. Apart from protein biomarkers, Scumaci et al. [183] explored miRNAs in plasma from 13 BrS patients and 10 healthy donors. MiRNA profiling showed 17 downregulated and 11 upregulated targets. Taqman assay confirmed that *miR-320b* and *miR-92a-3p* were significantly increased and *miR-425-5p* was considerably decreased in BrS plasma samples compared to controls. Notably, the miRNA alterations were similar in individuals who were either positive or negative for *SCN5A* variants.

These reports indicate that ncRNAs, miRNAs in particular, may not only act as mediators of BrS via coding genes but also act as potential biomarkers—diagnostic, prognostic, predictive, and/or therapeutic.

## 3. Discussion and Conclusions

Table 1 describes noncoding RNAs, noncoding genomic variants, Brugada Syndrome-related genes and corresponding ion channels. A recent systematic review showed that most countries globally do not report autopsy rates in either all-cause deaths or sudden deaths [186]. The majority of unexplained causes of SCD are attributed to sudden fatal arrhythmias [5]. BrS is a major SCD killer and definitive mutations have not been identified in the majority of the BrS patients [60,187,188]. The incomplete penetrance for those with identified mutations indicates that more than just that one gene may play a major role in the disorder. Both incomplete penetrance and variable expressivity are thought to be caused by factors including variants in regulatory regions, which include noncoding segments [189]. Several of the identified genes have been shown to be mediated by ncRNAs. More such findings can help develop a comprehensive transcriptomic network of how coding and ncRNAs interact to coordinately influence the genotype and phenotype of BrS [82]. The prospective BrAID multicenter clinical study involving the integration of machine learning algorithms and transcriptomics is expected to shed new light on mechanisms of type 1 BrS [190]. Future studies need to also focus on the early detection of noncoding variants and ncRNAs in the prevention of life-threatening arrhythmias and SCDs in BrS. While the roles of ncRNA-mediated regulation of stem cells and stem cell-derived/secreted ncRNAs need special attention in future BrS studies, iPSC-derived cardiac differentiated cells have been well-utilized in the study of BrS.

Given the relatively rare nature of the syndrome and challenges in obtaining human samples to study the disease, human in vitro models such as hiPSC-CMs offer huge benefits not only in mechanistic understanding but also in the development of novel diagnostic and therapeutic tools for the BrS community. HiPSC-CMs from BrS patients without identified mutations have been shown not to exhibit clear cellular electrophysiological abnormalities. One of the factors attributed to this observation was the involvement of possible noncoding genetic variants that can alter the expression of the known BrS-associated genes [191]. In addition, ajmaline can block both depolarization and repolarization of hiPSC-CMs; however, a more refined integrated tissue model was recommended to elicit differences between BrS patients and controls [155]. This raises the need for more 3D multicellular engineered human heart tissue models tailored for BrS phenotypes. This will enhance ncRNA research in vitro and offer translational findings potentially worthy of clinical trials.

With the advent of RNA-based vaccines for the COVID-19 pandemic, the awareness of RNA-based therapeutics has further improved. Multiple nucleic acid and RNA-based therapies have been approved by the U.S. Food and Drug Administration and/or European Medicines Agency, and ncRNA-based clinical trials are also ongoing [192,193,194,195,196,197,198]. This opens up the possibility for novel ncRNA therapeutics for BrS. These may be in the form of mimics to activate or inhibitors to block miRNA expression profiles [139,142]. Further research into the role of ncRNAs and stem cell biology in BrS will help develop novel diagnostic and therapeutic strategies for earlier detection and prevention of SCD.

Drug-induced adverse complications pose major life-threatening risks for BrS patients [28]. A recent open-label, multicenter, controlled, cluster-randomized crossover implementation study boosted the optimism around personalized medicine [199,200]. Using a 12-gene pharmacogenetic panel, the authors showed that genotype-guided treatment significantly reduced the incidence of clinically relevant adverse drug reactions. Furthermore, this was feasible across seven countries and diverse healthcare system organizations and settings. This further encourages the need for identifying all possible BrS genetic factors, especially in the ubiquitous noncoding compartment. Such efforts would also help find better diagnostic and therapeutic solutions for BrS, which causes innumerable instant and unanticipated deaths.

## Figures and Tables

**Table 1:** Noncoding RNAs, noncoding genomic variants, Brugada Syndrome-related genes and corresponding ion channels.

Noncoding Variant/RNA	Noncoding RNA Type	Associated Gene	Associated Ion Channel
*PCAT14*	Long noncoding RNA	*-*	-
Mitochondrial *tRNA*	Transfer RNA	*-*	-
*miR-192-5p*	MicroRNA	*SCN5A*	Na^+^ channel
*miR-219*	MicroRNA	*SCN5A*	Na^+^ channel
*miR-1270*	MicroRNA	*SCN5A*	Na^+^ channel
*miR-200c*	MicroRNA	*SCN5A*	Na^+^ channel
*miR-423-3p*	MicroRNA	*-*	-
*miR-223-3p*	MicroRNA	*-*	-
*miR-23a-3p*	MicroRNA	*-*	-
*miR-145-5p*	MicroRNA	*-*	-
*miR-585-3p*	MicroRNA	*-*	-
*miR-320b*	MicroRNA	*-*	-
*miR-92a-3p*	MicroRNA	*-*	-
*miR-425-5p*	MicroRNA	*-*	-
*rs41310749*	-	*SCN5A*	Na^+^ channel
*rs41310239*	-	*SCN5A*	Na^+^ channel
*rs6801957*	-	*SCN10A*	Na^+^ channel
*rs10428132*	-	*SCN10A*	Na^+^ channel
*6q22* region	-	*HEY2/NCOA7*	-
